# P-2109. Culture-Free Identification of Microbes in Seconds Directly from Clinical Samples using the MasSpec Pen Technology

**DOI:** 10.1093/ofid/ofae631.2265

**Published:** 2025-01-29

**Authors:** Rachel Downey, Manoj Kumar, Lindsey M Kirkpatrick, Sarmistha Bhaduri Hauger, Coreen Johnson, James Dunn, Faith Jackobs, Michael Keating, Livia Eberlin

**Affiliations:** Dell Children's Medical Center of Central Texas, Austin, Texas; Baylor College of Medicine, Dept. of Surgery, Houston, Texas; Riley Hospital for Children-IU Health, Indiana University School of Medicine, Division of Pediatric Infectious Diseases, Indianapolis, Indiana; Dell Children's Medical Center; Dell Medical School at the University of Texas at Austin, Austin, Texas; Texas Children's Hospital, Dept. of Pathology, Houston, Texas; Texas Children's Hospital, Houston, TX; Baylor College of Medicine, Dept. of Surgery, Houston, Texas; Baylor College of Medicine, Dept. of Surgery, Houston, Texas; Baylor College of Medicine, Dept. of Surgery, Houston, Texas

## Abstract

**Background:**

Broad spectrum antibiotics are often used empirically in cases of suspected invasive infection requiring surgical intervention while awaiting results of conventional testing (cultures and molecular tests). Rapid and accurate diagnosis of the etiologic pathogens is critical to allow for selection of targeted antibiotics and improve outcomes for patients.

We present current results of a large, multi-center clinical study using the MasSpec Pen (MSPen) technology to characterize the metabolic profiles of clinically relevant microbes in culture isolates and apply the technology to culture-independent identification of infectious agents directly in clinical samples.

**Figure 1.**
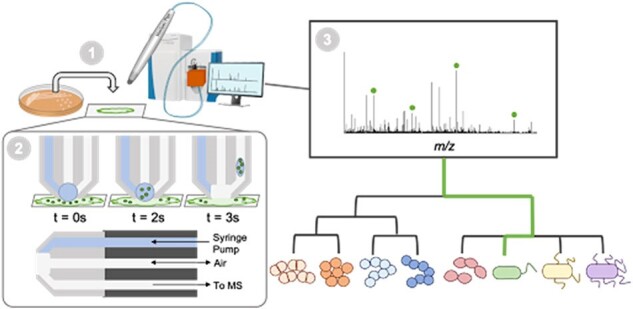
Schematics showing the key operating steps and results when using the MasSpec Pen to perform analysis and identification of bacteria.

**Methods:**

The MSPen allows direct sample analysis using a solvent droplet followed by immediate mass spectrometry analysis, with total acquisition time of ∼15 seconds (fig 1). In this study 785 samples (635 isolates and 150 clinical specimens) were analyzed using this technology to create distinct metabolic profiles to differentiate bacterial pathogens. 80% of data were used as a training set to develop such profiles; 20 percent of data were used as a test set to identify bacterial infection.

**Fig 2.**
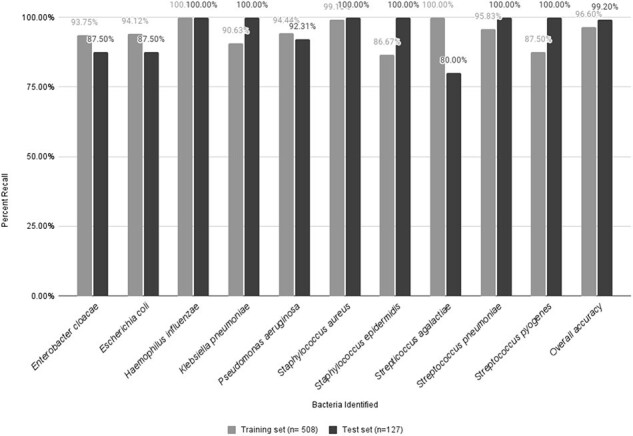
Species identification in pure isolates

**Results:**

We identified over 400 bacterial metabolites and lipids in 10 different microbial species and compiled a unique metabolic profile for each that was used to directly identify specific microbes in cultures and clinical specimens.

Among culture isolates, our statistical classifiers were able to achieve 99% accuracy for Gram-typing, and 99% accuracy among 10 bacteria in the test set (fig 2).

Using species-specific metabolites and lipids, we were able to identify *Pseudomonas aeruginosa* directly from 3 infected specimens and *Staphylococcus epidermidis* directly from infected bone.

**Conclusion:**

Our results show the incredible promise that direct MSPen analysis has for rapid, culture-independent identification of bacteria from patient tissues. We are working to build classifiers to differentiate bacteria by specific m/z values to improve identification in tissue samples.

**Disclosures:**

Manoj Kumar, PhD, LSE is an inventor in patents related to the MSPen technology owned by University of Texas Austin and Baylor College of Medicine. LSE is a co-founder,: LSE is an inventor in patents related to the MSPen technology owned by University of Texas Austin and Baylor College of Medicine. LSE is a co-founder, Lindsey M. Kirkpatrick, DO, PhD, Thermo Fisher Scientific: Honoraria Michael Keating, PhD, MS Pen Technologies: Advisor/Consultant Livia Eberlin, PhD, Merck: Honoraria

